# The Structural Features of Trask That Mediate Its Anti-Adhesive Functions

**DOI:** 10.1371/journal.pone.0019154

**Published:** 2011-04-29

**Authors:** Danislav S. Spassov, Deepika Ahuja, Ching Hang Wong, Mark M. Moasser

**Affiliations:** 1 Department of Medicine, University of California San Francisco, San Francisco, California, United States of America; 2 Helen Diller Family Comprehensive Cancer Center, University of California San Francisco, San Francisco, California, United States of America; Heart Center Munich, Germany

## Abstract

Trask/CDCP1 is a transmembrane protein with a large extracellular and small intracellular domains. The intracellular domain (ICD) undergoes tyrosine phosphorylation by Src kinases during anchorage loss and, when phosphorylated, Trask functions to inhibit cell adhesion. The extracellular domain (ECD) undergoes proteolytic cleavage by serine proteases, although the functional significance of this remains unknown. There is conflicting evidence regarding whether it functions to signal the phosphorylation of the ICD. To better define the structural determinants that mediate the anti-adhesive functions of Trask, we generated a series of deletion mutants of Trask and expressed them in tet-inducible cell models to define the structural elements involved in cell adhesion signaling. We find that the ECD is dispensable for the phosphorylation of the ICD or for the inhibition of cell adhesion. The anti-adhesive functions of Trask are entirely embodied within its ICD and are specifically due to tyrosine phosphorylation of the ICD as this function is completely lost in a phosphorylation-defective tyrosine-phenylalanine mutant. Both full length and cleaved ECDs are fully capable of phosphorylation and undergo phosphorylation during anchorage loss and cleavage is not an upstream signal for ICD phosphorylation. These data establish that the anti-adhesive functions of Trask are mediated entirely through its tyrosine phosphorylation. It remains to be defined what role, if any, the Trask ECD plays in its adhesion functions.

## Introduction

Cell adhesion to the extracellular matrix (ECM) is an important function in metazoans. Adhesion to matrix forms structural anchorage points that function to map the architectural layout of cells within a tissue framework, while linking the intracellular actin cytoskeleton with the extracellular matrix (ECM), providing structural stability at the tissue level. Cell adhesion also mediates intracellular signaling functions that regulate aspects of cell behavior cell growth, survival, proliferation, and migration. The regulation of cell adhesion is a highly coordinated process involving heterodimeric integrin receptors and many other classes of cytoplasmic and membrane proteins. Upon integrin engagement to the ECM and clustering of integrins at sites of cell adhesion to matrix, macromolecular complexes are assembled in association with the intracellular tails of activated integrins [1], [2], [3]. Within these focal adhesions, focal adhesion kinase (FAK) is activated by autophosphorylation at tyrosine 397, creating a binding site for the Src SH2 domain [1], [4]. Upon binding to FAK, Src is activated and phosphorylates a number of additional tyrosine residues on FAK, creating additional binding sites for SFKs and other proteins. Activated Src also phosphorylates a number of additional cytoskeletal proteins including paxillin and p130Cas and proteins involved in regulating the RhoA, Rac1, and Cdc42 GTPases [5]. These events function to stabilize focal adhesions, generating a force-induced mechanical link with the actin cytoskeleton, and regulate the surrounding membrane dynamics. While our understanding of adhesion complex formation has greatly evolved in recent years, our understanding of how adhesion complexes are disrupted remains largely primitive. The mechanisms that promote the disruption of cell adhesion are of particular interest in cancer biology, specially for the study of invasion and metastasis. We have been studying a cell surface protein that is a negative regulator of cell adhesion.

Trask (*Tr*ansmembrane and *A*ssociated with *S*rc *K*inases) is a membrane glycoprotein whose functions are not yet well understood. Trask (aka CDCP1, SIMA135, gp140) has been independently identified by several groups pursuing different lines of study. One group identified it as a transcript expressed in colon cancers [6]; our group identified it as a Src substrate phosphorylated during mitotic detachment [7]; another group identified it as an adhesion related protein tyrosine phosphorylated following exposure to suramin or vanadate [8], and another group identified it in a search for surface antigens associated with a more metastatic cancer phenotype [9]. Trask/CDCP1 is a 140 kD transmembrane glycoprotein with a large extracellular domain (ECD) containing CUB domains, and a smaller intracellular domain (ICD) containing five tyrosines [7], [10]. The tyrosine phosphorylation of Trask is tightly regulated and reciprocally linked with the state of cell adhesion. The tyrosine phosphorylation of Trask in cultured cells occurs when cells are induced to detach by trypsin or EDTA, or seen spontaneously during mitotic detachment [7], [10] [8]. In overexpression studies we found that the overexpression of Trask leads to the loss of cell adhesion and a detached phenotype [7]. Trask is widely expressed in human epithelial tissues, but its phosphorylation is only seen in mitotically detached or shedding cells, consistent with its role in the negative regulation of cell adhesion [10]. The phosphorylation of Trask is seen in many cancers, including some pre-invasive cancers as well as in invasive tumors and in tumor metastases [11]. The functional implications of Trask phosphorylation in tumors is currently unknown.

Since Trask has little homology with other genes or gene families, its functions have been difficult to predict and much more experimental evidence from biochemical and biological studies are necessary to begin to understand its functions. Several studies mentioned previously point to a role in the regulation of cell adhesion. But it remains unclear whether this adhesion function is mediated through tyrosine phosphorylation or through the functions of the extracellular domain, or a more complex outside-in or inside-out signaling function involving both the intracellular and extracellular domains. The functions of the Trask ECD are largely unknown. CUB domains are extracellular protein-protein interaction modules, typically found in many proteases. Indeed Trask is stably associated with the CUB domain containing membrane protease MT-SP1 and is a proteolytic substrate of MT-SP1 [7]. Trask is cleaved by other serine proteases including trypsin and plasmin as well and this accounts for the expression of larger (140 kD) and smaller (approximately 80 kD) forms of Trask that are typically observed in different ratios in different cell types [11], [12].

Some investigators have suggested that cleavage of the Trask/CDCP1 ECD results in phosphorylation of its ICD [12]. The evidence that has promoted this suggestion is that cells treated with trypsin simultaneously undergo Trask cleavage, cell detachment, and Trask phosphorylation [8], [10], [12]. Although the cleavage and phosphorylation of Trask/CDCP1 may occur simultaneously in some circumstances, the cleavage of the ECD does not appear to be required for phosphorylation of the ICD as the phosphorylation of Trask is also seen with EDTA-induced cell detachment without any increase in Trask cleavage beyond the basal state [11]. Clearly, we need more experimental studies to explore the functional relationship between the Trask ECD and ICD, particularly as it pertains to its role in the regulation of cell adhesion. In the current study we have undertaken a structure-function analysis of Trask in order to determine whether its anti-adhesive functions reside within the ECD or ICD and whether this involves transmembrane signaling. The data reveals that the anti-adhesive functions of Trask are directly mediated through the tyrosine phosphorylation of its ICD and the ECD is dispensible for its tyrosine phosphorylation or for the inhibition of cell adhesion when phosphorylated.

## Results

**Figure 1:**
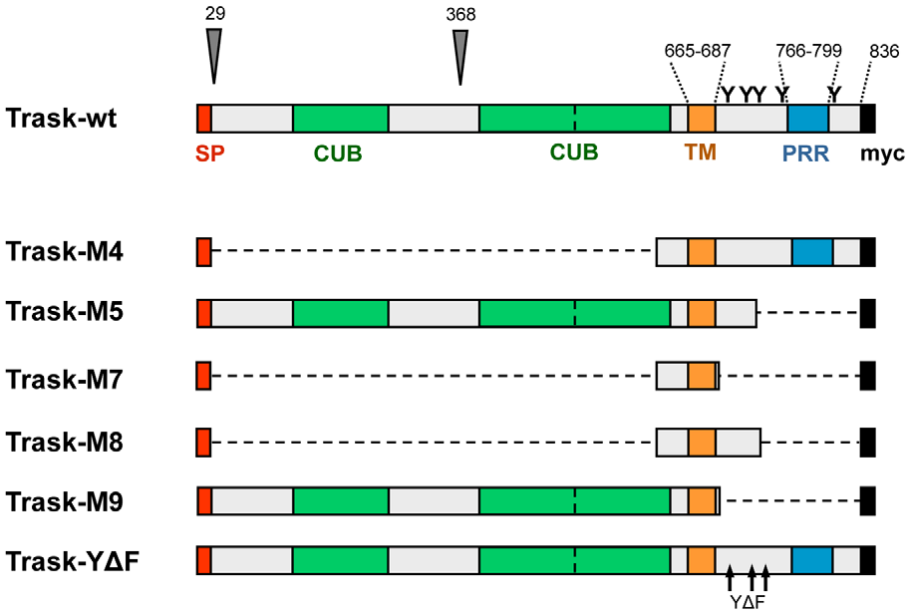
Generation of Trask mutant constructs. Schematic representation of the Trask mutant constructs. All constructs contain the signal peptide and transmembrane regions for proper membrane localization and an in-frame c-terminal myc tag for easy identification. The triangles indicate the naturally occurring cleavage sites after the signal peptide and at Arg368 of the extracellular domain. Abbreviations indicate the signal peptide (SP), CUB domains, transmembrane region (TM), proline-rich region (PRR), and myc tags. The extracellular region homology to CUB domains is low, and the precise region and number of CUB domains is somewhat speculative. The location of the 5 intracellular tyrosines are indicated by Y.

**Figure 2:**
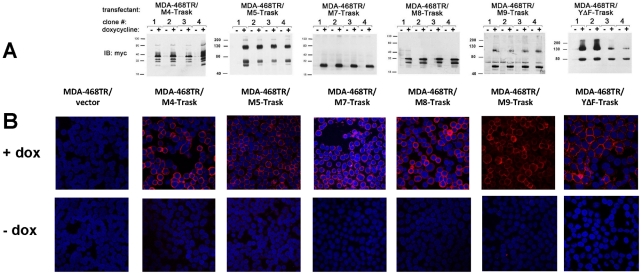
Inducible expression of Trask mutants in MDA-468 cells. **A**) MDA-468TR cells were engineered to inducibly overexpress the M4, M5, M7, M8, and M9 and YΔF Trask mutants in response to doxycycline treatment. For each construct four clones were studied to account for clonal variability. The dox-inducible expression of the M4, M5, M7, M8, and M9 and YΔF Trask mutants are shown by anti-myc immunoblots. The myc immunoblots for the wt-Trask over-expressing cells were previously published [7]. These induction blots are representative of two experiments done with the entire clone set, and with 4 experiments done with selected Trask-M4 and Trask-YΔF clones. **B**) The membrane localization of each of the induced constructs was verified following doxycycline induction by anti-myc staining of fixed cells and immunofluorescence microscopy.

We previously established MDA-468 cells with tet-inducible overexpression of Trask and found that overexpression of Trask is associated with the loss of cell adhesion and continued growth in a rounded suspended state [7]. To determine the structural features of Trask that are required for this anti-adhesive function, we generated a series of Trask deletion mutants that lack residues within the ICD or ECD. The structure of these mutants, the specific regions deleted, and the c-terminal myc tag are depicted schematically in Figure 1. Each of these constructs were stably transfected into MDA-468 cells previously established to express the tet repressor (MDA-468TR). For each Trask mutant, multiple clones were expanded and analyzed and the dox-inducible expression of the Trask mutants confirmed by anti-myc immunoblotting (Figure 2A). The M5 and M9 mutants retain the entire ECD, while the M4, M7, and M8 mutants have deletions of nearly the entire ECD. The M5 and M9 mutants are expressed as both larger and smaller forms consistent with the partial proteolytic cleavage of their ECDs, as occurs with the wildtype Trask. The functions of the intracellular domain are not required for the cleavage of the ECD as the M9 mutant, lacking the entire ICD, undergoes proteolytic cleavage. The partial nature of the proteolytic cleavage is similar to that seen with tet-inducible overexpression of the wildtype Trask which we have previously shown [7]. While there are differences in the relative expression of cleaved and uncleaved products of the wildtype and mutant Trask constructs, these could be due to the different stability of these proteins.

All constructs localize to the cell membrane as shown by fluorescence microscopy of anti-myc immunostained cells following doxycycline treatment (Figure 2B). Therefore neither the ECD or the ICD functions are required for membrane localization. The YΔF mutant also localizes to the membrane indicating that phosphorylation is also not required for membrane localization. The signal peptide and transmembrane regions were preserved in all constructs, since they are known to be required for proper membrane localization of type I transmembrane proteins.

**Figure 3:**
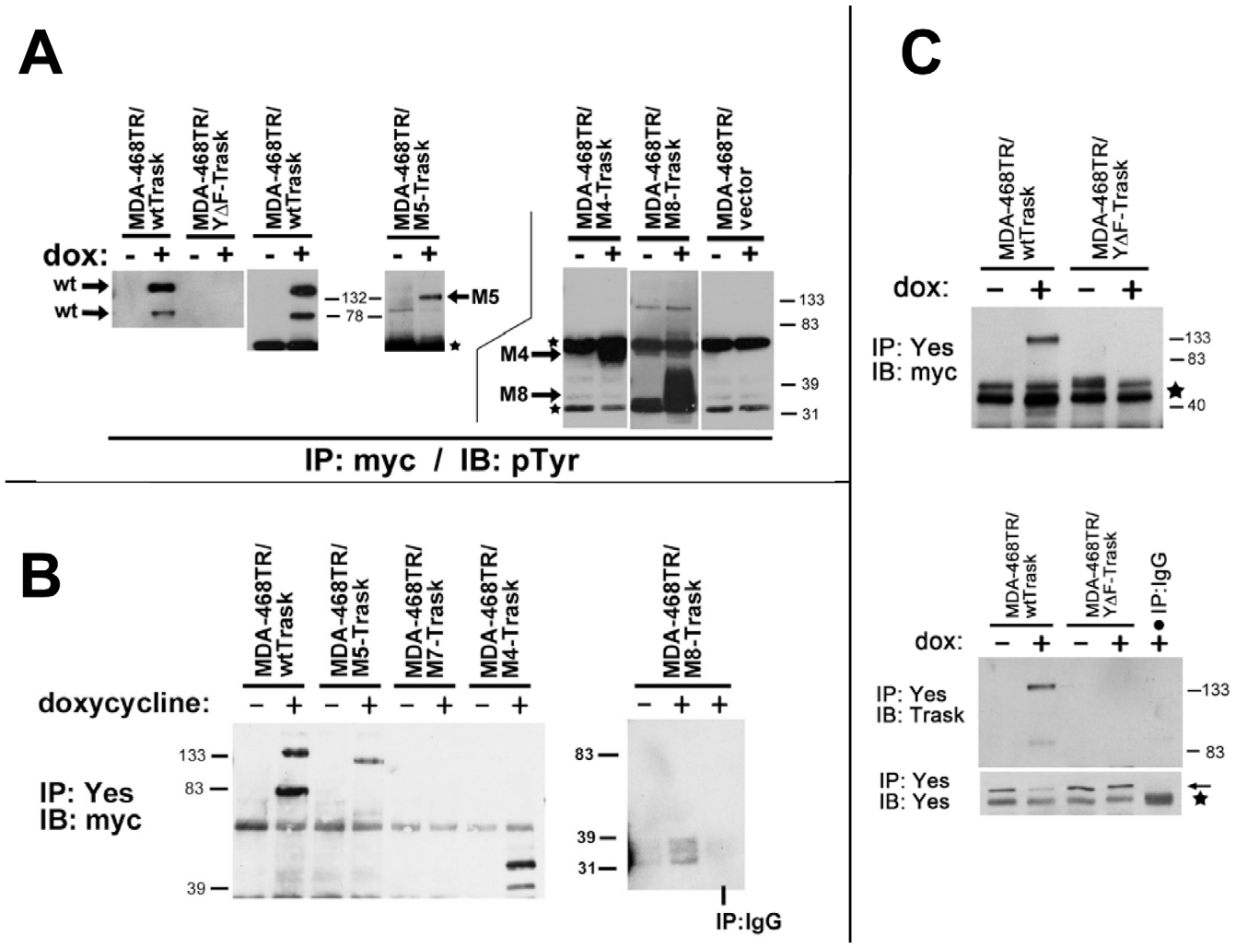
Signaling properties of Trask mutants. **A**) The induced constructs were immunoprecipitated by anti-myc antibodies from the engineered transfectants cell types and vector control cells and immunoblotted with pTyr antibodies. The lysates from the larger size constructs (wt, YΔF, M5) were run on lower % gels and the smaller size constructs (M4, M8) were run on higher % gels for optimal separation and identification. The bands corresponding to the various constructs are indicated by arrows. The starts indicate the immunoglobulin chains. These blots are representative of multiple experiments (4X for wt, 4X for YΔF, 2X for M5, 2X for M4, 3X for M8, and 2X for vector control). **B**) Anti-yes immune complexes were obtained from each of the mutant cell types and immunoblotted using anti-myc antibodies to detect the co-immunoprecipitation of the transfected constructs. The M8 mutant Trask protein appears as 3 bands likely due to the significant effect of differential phosphorylation on the gel migration of this small construct. These blots are representative of multiple experiments (5X for wt, 1X for M5, 2X for M7, 2X for M4, and 3X for M8). **C**) The interaction of the YΔF mutant with Yes was similarly assayed as above. The anti-myc immunoblot shows that the Yes-Trask interaction is lost in the YΔF mutant. The immunoblots were redone using anti-Trask antibodies, again confirming that the Yes-Trask interaction is lost in the YΔF mutant. The stars indicates the immunoglobulin chains. The arrows indicate the Yes protein. These blots are representative of three experiments.

The state of phosphorylation of each expressed construct was determined by anti-myc immunoprecipitation followed by phosphotyrosine immunoblotting. When overexpressed, wildtype Trask undergoes constitutive tyrosine phosphorylation (Figure 3A). The reasons for this are not clear, but are likely due to the saturation of dephosphorylation mechanisms. The M4, M5, and M8 constructs that contain ICD regions also undergo tyrosine phosphorylation (Figure 3A). The M8 mutant, which retains only 3 of the 5 intracellular tyrosines, also undergoes tyrosine phosphorylation. There is no detectable tyrosine phosphorylation of the M7 mutant which lacks an intracellular domain (Figure S1) or the YΔF mutant which lacks critical tyrosines within the ICD (Figure 3A).

We previously described that Trask is physically associated with Src kinases, in particular with Yes [7]. The presence of this interaction was determined for all the Trask mutants. The M4, M5, and M8 constructs that contain ICD regions and phosphorylated tyrosines interact with Yes (Figure 3B). The mutants that lack the ICD or the three intracellular tyrosines fail to interact with Yes (Figure 3B). The YΔF mutant also fails to interact with Yes (Figure 3C). Conclusions cannot be drawn regarding any quantitative differences among the mutants with regards to tyrosine phosphorylation or with regards to interaction with Yes. This is because the constructs have different expression characteristics, likely due to different protein half lives, precluding direct comparative analyses of their phosphorylation or interaction levels.

**Figure 4:**
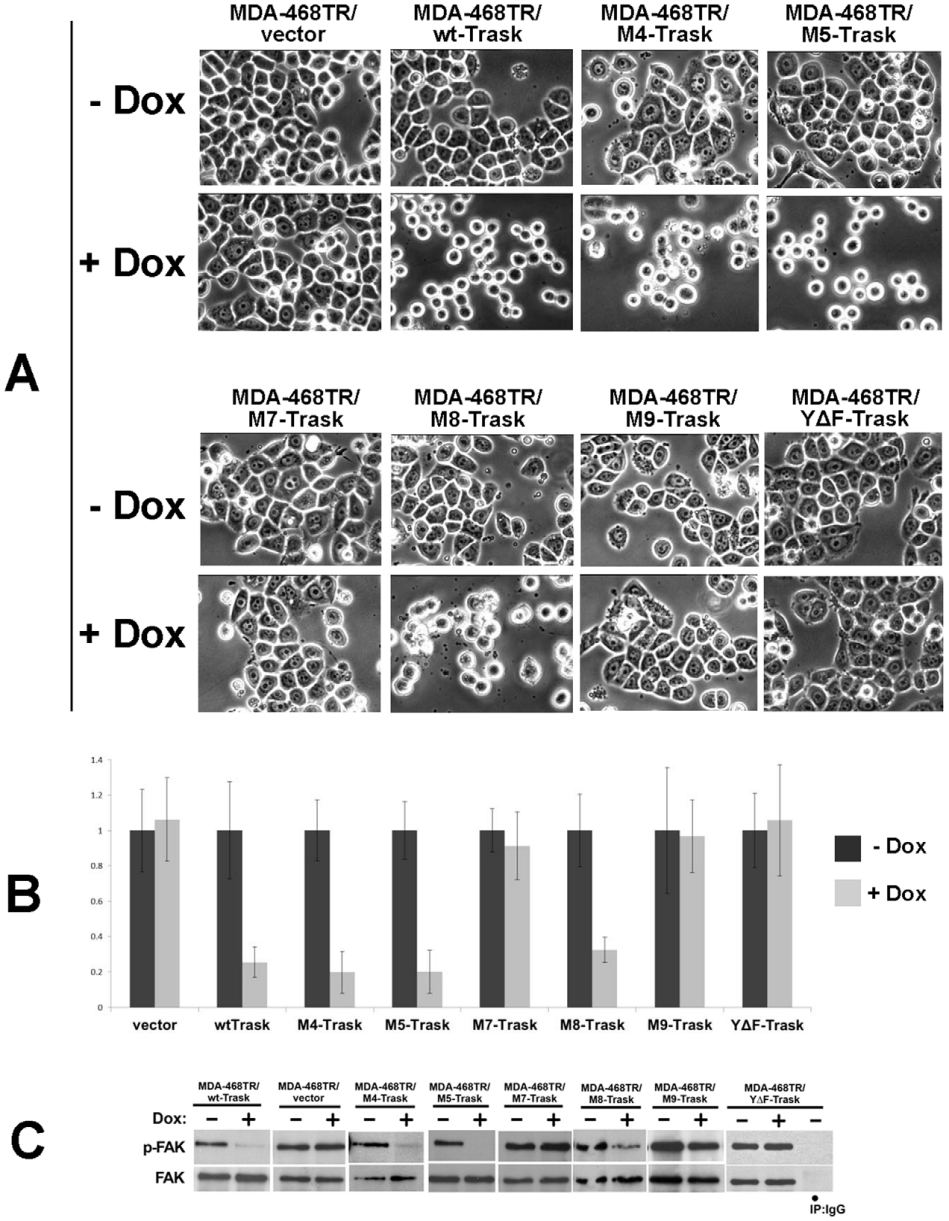
Effects of Trask mutants on cell adhesion. **A**) The indicated cell types were plated in 6-well plates in the presence or absence of 1 ug/ml doxycycline and the following day imaged under phase contrast microscopy. These views are representative of multiple fields of view (shown in Figure S2) and representative of multiple independent experiments (>10X for wt, 5X for M4, 3X for M5, 3X for M7, 3X for M8, 3X for M9, and 5X for YΔF). **B**) The indicated cell types were treated with 1 ug/ml doxycycline or control and the following day seeded into 96-well plates in triplicate wells and the adherent cell population was quantified by crystal violet staining as described in methods. Results were normalized to non-induced controls, and averaged across the triplicate wells as well as across three independent experiments done on different days (n = 9), in different clones, with the indicated SEM. The reduced cell adhesion following dox induction is statistically significant for WT-Trask (p<0.0001), M4-Trask (p<0.0001), M5-Trask (p<0.0001), and M8-Trask (p<0.0001) cell types. **C**) The indicated cell types were treated with 1 ug/ml doxycycline or control overnight and the following day total cell lysates were used to determine focal adhesion signaling. FAK phosphorylation was done by anti-FAK immunoprecipitation followed by anti-phosphotyrosine immunoblotting. These blots are representative of multiple experiments (5X for wt, 5X for vector control, 2X for M4, 2X for M5, 2X for M7, 2X for M8, 2X for M9, and 5X for YΔF).

Next we determined the effect of each of the Trask mutants on the anti-adhesive function of Trask. When overexpressed, Trask is constitutively phosphorylated (shown above) and inhibits cell spreading and adhesion [7]. The effects on cell adhesion were qualitatively and quantitatively assessed following doxycycline induction. The M4, M5, and M8 mutant constructs retain the ability to inhibit cell adhesion similar to wild type Trask (Figure 4A,B). After doxycycline induction, these cells have 10% (wt), 9% (M4), 9%(M5), and 28%(M8) of the number of adherent cells seen in their uninduced controls. On the other hand the M7, M9, and YΔF mutants fail to inhibit cell adhesion showing cell adhesion after doxycycline induction that is 96% (M7), 113% (M9), and 123% (YΔF) of their uninduced controls. These results are consistent among four different clones of each transfectant and therefore are not due to clonal variation. Data obtained from additional clones, with microscopic images from several additional fields and magnifications, and quantitative adhesion assays from additional clones are shown in Figure S2. When doxycycline induction is initiated in detached cells at the time of plating, cells fail to adhere. When the induction is initiated in fully adherent cells, there is also an inhibition of cell adhesion, although the loss of cell adhesion occurs more gradually. The induction of Trask overexpression results in the dephosphorylation of focal adhesion kinase, consistent with the disruption of cell adhesion and dismantling of focal adhesions (Figures 4C and S3). This is seen with the wildtype Trask as well as with the M4, M5 and M8 mutants. The M7 and M9 mutants that lack the ICD fail to inhibit cell adhesion. The YΔF mutant also fails to inhibit cell adhesion or inhibit focal adhesion signaling.

**Figure 5:**
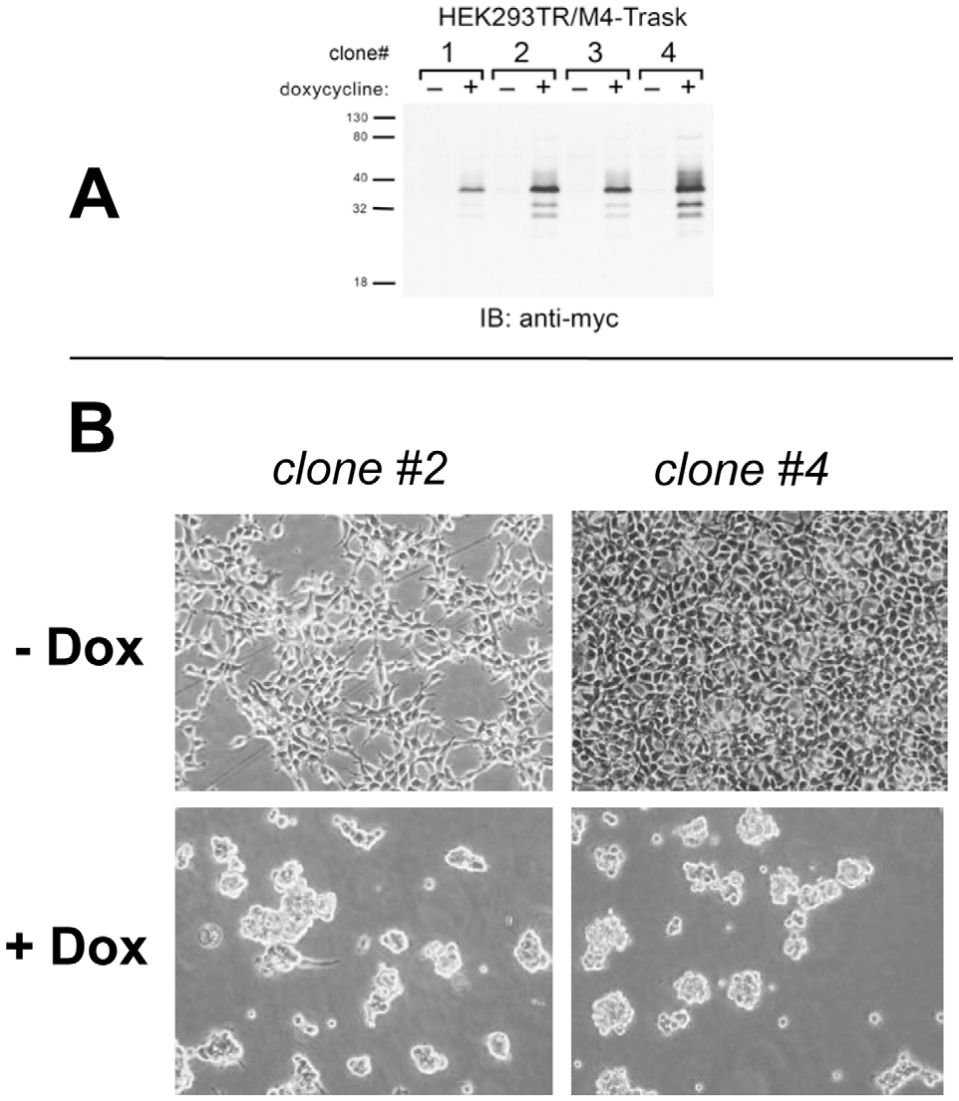
Effects of Trask phosphorylation in HEK293 cells. **A**) HEK293TR cells were engineered to express the M4-Trask mutant in a tet-inducible fashion. The doxycycline inducible expression of M4-Trask is shown here for four different clones. This blot is representative of two independent experiments. **B**) HEK293TR/M4-Trask cells were plated in the presence or absence of 1 ug/ml doxycycline and the following day observed under phase contrast microscopy. Although not obvious in these still photographs, many of the cell clumps in the doxycycline treated arm are loosely adhered or entirely in suspension. This experiment has been performed 4 times with similar results.

These data show that the anti-adhesive function of Trask are embodied within the ICD and are entirely dependent on the tyrosine phosphorylation of the ICD. The ECD appears to be dispensible for this function as shown by mutants that lack the ECD. It remains possible that the mutants lacking the ECD inhibit adhesion through a dominant negative mechanism involving the endogenous full length Trask and its ECD. To rule out this possibility we established HEK293 cells with tet-inducible expression of the M4 Trask mutant (Figure 5A). HEK293 cells have no detectable endogenous expression of Trask, enabling us to test the anti-adhesive functions of the Trask ICD directly and definitively, ruling out a potential orthogonal role for the endogenous Trask ECD. Expression of the Trask M4 mutant in these cells leads to a profound inhibition of cell adhesion (Figure 5B). This confirms that the anti-adhesive functions of Trask are contained entirely within its ICD.

**Figure 6:**
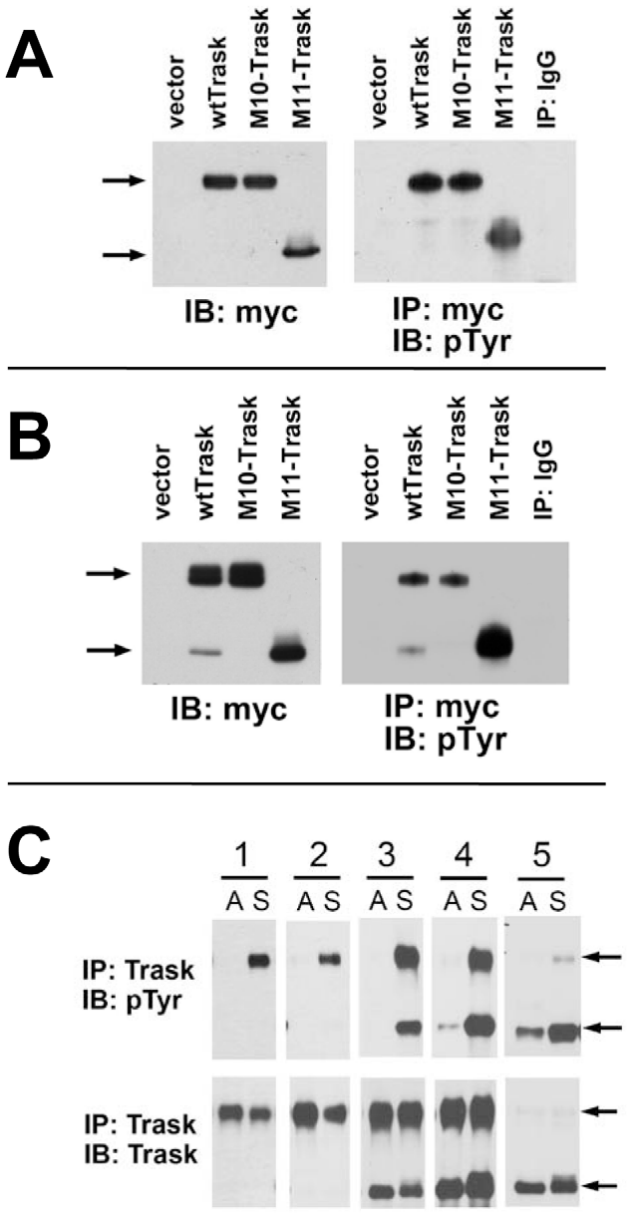
Trask cleavage and phosphorylation. **A,B**) HEK293 (A) or MDA-468 (B) cells were transiently transfected with the indicated mutant constructs or wildtype or vector controls and total cell lysates were assayed as indicated. These blots are representative of multiple experiments (shown in Figure S4). **C**) Cell lysates from five cell lines were harvested while adherent (A) or after 2 hours of culture in suspension (S) and immunoblotted as indicated. Lanes correspond to 1) L3.3 cells, 2) SW480 cells, 3) DLD1 cells, 4) PC3 cells, and 5) MDA-468 cells. Arrows indicate the larger uncleaved and smaller cleaved forms of Trask. These blots are representative of two experiments. The MDA-468 blot is representative of >5 independent experiments.

Although the ECD is not required for the anti-adhesive functions of Trask, it may still provide an outside-in signal to induce the phosphorylation of Trask, or it may function to inhibit Trask phosphorylation until it is cleaved, and thereby function indirectly in the regulation of the anti-adhesive functions of Trask. To interrogate this, we generated a mutant Trask construct that is resistant to cleavage. We have previously identified the site of cleavage by MT-SP1 in MDA-468 cells as arginine 368 [7]. To generate an MT-SP1 resistant construct we mutated this critical arginine to aspartate. The R368D Trask mutant (named M10) was confirmed to be resistant to cleavage in transient transfection assays. We also generated a Trask mutant that is identical to the membrane-bound proteolytic product of Trask cleavage at R368 (named M11). When expressed in MDA-468 cells or in HEK293 cells, both the cleavage-resistant M10 mutant and the cleavage-product M11 mutant undergo tyrosine phosphorylation very efficiently (Figure 6A,B). Although there is evidence that Trask can also be cleaved at sites other than R368 [12], this does not impact the interpretation of our experiment, since the M10 (R368D) mutant is clearly fully resistant to cleavage in our experiments. The cleavage-product M11 mutant appears more abundantly phosphorylated than wildtype or M10 Trask. Trask cleavage is clearly not required for its phosphorylation since the uncleaved wildtype Trask and the cleavage-resistant M10 Trask mutant readily undergo tyrosine phosphorylation. The absence of a relationship between Trask cleavage and its phosphorylation is easily evident when comparing cell lines. There are cell types in which Trask is predominantly uncleaved, and wherein Trask undergoes detachment-induced phosphorylation without any cleavage (Figure 5C). There are also cell lines wherein Trask is expressed as a blend of cleaved and uncleaved forms, and both forms undergo detachment-induced phosphorylation (Figure 5C). There are also cell types wherein Trask is predominantly cleaved wherein it is this form that undergoes detachment-induced phosphorylation (Figure 5C). Therefore, the phosphorylation of Trask is tightly regulated by cell anchorage as we have previously described [10]. The proteolytic cleavage of Trask does not appear to be a significant regulator of its phosphorylation. The mechanisms that regulate and determine the extent of cleavage of Trask are currently unknown.

## Discussion

Trask/CDCP1 is a transmembrane protein whose functions are not yet well understood. It has been identified in several independent lines of research as a cancer-associated protein and proposed to have functions relevant to tumor progression. But the data sets that have emerged in the investigation of its role in cancer progression have been mixed and partly conflicting. Some studies, mostly looking at Trask RNA expression, have suggested that its expression is significantly increased in cancers Scherl-Mostageer, 2001 #90; Miyazawa, 2010 #238; Perry, 2007 #82; Uekita, 2008 #78}. A limited number of studies appear to suggest a function in cancer invasion or metastasis [9], [13]. But in larger immuno-histochemical studies it has become clear that Trask is widely expressed in most normal epithelial tissues, quite abundantly in some, and similarly widely expressed in epithelial cancers without significant evidence of overexpression [10], [11]. There is immuno-histochemical evidence showing reduced Trask/CDCP1 expression in some cancers and biochemical data showing that a reduction or loss of expression in some cancer cells due to promoter hypermethylation [14], [15]. Attempts to correlate the level of expression of Trask in human tumors with clinical outcome has also produced conflicting data sets as both poorer and better prognoses have been linked with higher Trask expression [16], [17], [18].

Clearly our understanding of Trask function is still evolving, and awaits more insight into its biochemical and cellular functions. One function that is clear is a function in the regulation of cell adhesion. In normal cells, the phosphorylation of Trask is exclusively seen in the unanchored state and its overexpression inhibits cell adhesion [7], [8], [10]. Some have suggested that phosphorylation of Trask is induced by the proteolytic cleavage of its ECD [8], [12]. This would suggest an outside-in signaling function. To more directly investigate what functions of Trask mediate its anti-adhesive effects, we conducted a structure-function analysis of Trask using a series of mutant constructs. This analysis reveals that the anti-adhesive functions of Trask are specfically due to its tyrosine phosphorylation. Its ECD is dispensible for the inhibition of cell adhesion as mutant constructs lacking the ECD are fully capable of inhibiting cell adhesion. The ECD also does not indirectly regulate cell adhesion through the regulation of Trask phosphorylation since both the wildtype full length Trask or a cleavage-resistant Trask full length Trask mutant efficiently undergo tyrosine phosphorylation. Since we find higher phosphorylation of cleaved Trask in MDA-468 cells, it remains possible that cleaved Trask is more efficient as a substrate for phosphorylation. Therefore the proteolytic cleavage of Trask may provide one input that influences its phosphorylation state, but this is a quantitative difference and may have contextual relationships that we don't yet understand. The mechanisms that regulate the proteolytic cleavage of Trask are not currently understood. As such the basis for the varying degrees of Trask cleavage seen among different cell lines remains to be explained. Even the same cell line shows modest changes in the degree of Trask cleavage in different experiments without a methodological basis or an obvious phenotypic difference.

These studies establish that the anti-adhesive functions of Trask are specifically due to the tyrosine phosphorylation of its ICD. Therefore the functions of the Trask ECD remain largely unknown at this time, awaiting hypotheses regarding what its functions may be, and studies designed to specifically test such hypotheses.

## Materials and Methods

### Cell culture and reagents

MDA-468 and HEK293 cells were obtained from the American Type Culture Collection and grown in DMEM media supplemented with 10% heat-inactivated tetracycline-free fetal bovine serum, and 100 U/ml penicillin, 100 µg/ml streptomycin, 4 mM glutamine, and incubated at 37 C in 5% CO2. To force cells into suspension, cells were washed in PBS and exposed to a 2 mM solution of EDTA in Hank's buffer, and when fully detached, were cultured in growth media in ULC plates (Corning) for 2 hours. ULC plates are not permissive to cell adhesion. Antibodies were purchased from SantaCruz Biotechnology (anti-myc 9E10, anti-phosphotyrosine PY99, anti-FAK A-17) and Wako (anti-yes 1B7), and anti-Trask M19. Total cellular lysates were harvested in modified RIPA buffer (10 mM Na phosphate (pH 7.2), 150 mM NaCl, 0.1% SDS, 1% NP40, 1% Na deoxycholate, protease inhibitors and 1 mM sodium orthovanadate). For western blotting, 50 ug of each lysate was separated by SDS-PAGE, transferred to membrane, and immunoblotted using appropriate primary and secondary antibodies and enhanced chemoluminescence visualization. For immunoprecipitation studies, 300 ug of lysate was incubated overnight with specific antibodies, immune complexes collected by protein G-sepharose beads, washed, and the denatured complexes immunoblotted as above.

### Phase contrast microscopy

Phase contrast microscopy was performed using a Nikon TS-100F inverted microscope equiped with a Nikon D100 digital camera attached to the photo port. Images were imported into Photoshop software, converted to gray scale, and gamma adjusted for optimal representation.

### Adhesion assay

Adhesion was quantitatively assessed by a colorimetric assay using crystal violet (Sigma), a cytochemical stain that binds to chromatin. Cells were induced for 16 hrs with or without 1 *µ*g/ml doxycycline and subsequently plated at a density of 5×10^5^ cells/ml in a volume of 100 μl per well in 96 well plates. After two hours, wells were washed three times with PBS to remove unattached cells. Attached cells were fixed in cold methanol for 15 minutes, plates were then air dried and stained with 0.1% crystal violet in PBS for five minutes at room temperature. After removing the crystal violet solution, the plates were washed extensively, dried, and the stain released using 2% SDS in PBS. Stain intensity was quantified by spectrophotometry at 570 nm using a plate reader.

### Immunoblotting and immunofluorescent studies

Western analysis and immunoprecipitation were performed using horseradish perodixase linked secondary reagents and visualized by enhanced chemoluminescence. For immunolocalization studies cells were grown on fibronectin coated glass cover slips and fixed in 4% paraformaldehyde., and permeabilized in 0.1% triton-X. Fixed cells were incubated with 1∶100 dilution primary antibody and subsequently with rhodamine conjugated secondary antibodies and visualized under fluorescent microscopy at the appropriate excitation wavelengths.

### Vector constructs and transfections

The 2.5 kb human Trask cDNA ORF was cloned into the pcDNA4/TO/MycHis vector (Invitrogen) in-frame with the c-terminal myc and his tags under control of the CMV promoter containing tet-operator sequences as previously described [7]. The deletion mutants were generated using insertional and deletional PCR cloning techniques using the following primers and templates: M4 primers (deletion cloning from full length template, lacks AA 39–618): TTCAGCCTGGACGAGGATGTG and GCTTTCTCGTGGCAGAGCAATC; M5 primers (insertional cloning from full length template, lacks AA 754–837): CGAATTCGTCCCGGAGTCATGGCCG and CAGTCTCGAGTGAGCCGCTGGAATCCTGTAG; M7 primers (deletion cloning from M4 template, lacks AA 39–618, 692–841): GGGCCCTTCGAACAAAAACTC and CACACAGCAAATGATGAGCCC; M8 primers (deletional cloning from M5 template, lacks AA 39–618 and 754–837): TTCAGCCTGGACGAGGATGTG and GCTTTCTCGTGGCAGAGCAATC; M9 primers (deletional cloning from M5 template, lacks AA 692–841): GGGCCCTTCGAACAAAAACTC and CACACAGCAAATGATGAGCCC. The YΔF Trask mutant was generated from the wildtype Trask expression plasmid using the Stratagene Quick Change II site directed mutagenesis kit. Initially the tyrosines at position 707 was mutated to phenylalanine, and the product was subsequently used to mutate the tyrosines at positions 734 and 743 in a second site directed mutagenesis step. The M10 mutant was generated from the wildtype Trask by site directed mutagenesis to mutate arginine 368 to aspartate. The M11 mutant was generated by deletional PCR cloning using the primers TTCTGCCCCGCGCGGC and AAGTTTGTCCCTGGCTGTTTCGTGTGTCTAGAATCTCGGACC and lacks AAs 29–368. All constructs were fully sequenced and confirmed. To generate stable tet-inducible transfectants MDA-468TR cells or 293TR cells (both previously transfected with the pcDNA6/TR vector) were transfected with the desired constructs or the parent pcDNA4/TO/MycHis vector using lipofectamine 2000 and stable transfectants selected in Zeocin. Single cell derived clones from each construct were picked. Individual clones were picked and gene expression verified by induction with 1 *µ*g/ml doxycycline for 24 hours and anti-myc western blotting.

## Supporting Information

Figure S1 **Cell lysates from the indicated transfectant cell types were immunoprecipitated with anti-myc antibodies and immunoblotted with anti-phosphotyrosine antibodies.**
(PDF)

Figure S2 **On the following pages several different views are shown for each cell type with/without doxycycline induction.** The high magnification view is shown to best demonstrate morphologic characteristics while the low magnification view is shown to best demonstrate the generality of the adhesion phenotype across a larger cell population with much less selectivity due to the wider field of view. The microscopic images in this figure are from different clones than the ones shown in the main paper. This is to show consistency and account for effects related to clonal variability.
(PDF)

Figure S3 **The FAK phosphorylation data of** **Figure 4C** **was quantified by densitometry using the ImageJ program.** The analysis was done separately for each paired data set (+/− doxycycline) corresponding to the different transfected cell types and normalized with respect to the uninduced (-dox) state. The data was not normalized with respect to total FAK expression, as a direct effect of the induction on FAK expression cannot be ruled out.
(PDF)

Figure S4 **MDA-468 cells were transiently transfected with the pcDNA4 vector or vector expressing the wildtype, M10, or M11 Trask mutants.** Cell lysates were assayed as indicated. These are independent repeats of the experiment shown in the manuscript.
(PDF)
