# Supplementary material for: The Structural Features of Trask That Mediate Its Anti-Adhesive Functions
Source: PLoS One. 2011 Apr 29;6(4):e19154. doi: 10.1371/journal.pone.0019154 (PMC3084758; doi:10.1371/journal.pone.0019154)
Supplement: Figure S1 — Cell lysates from the indicated transfectant cell types were immunoprecipitated with anti-myc antibodies and immunoblotted with anti-phosphotyrosine antibodies. (PDF) [file pone.0019154.s001.pdf]

Figure S1

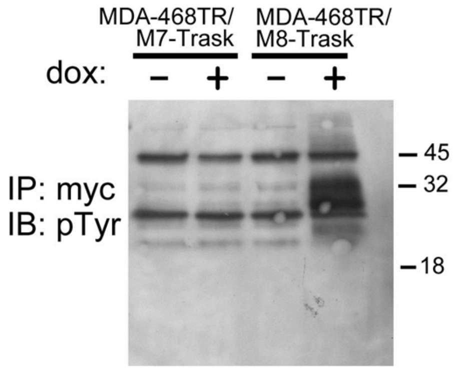

Figure S1: Cell lysates from the indicated transfectant cell types were immunoprecipitated with anti-myc antibodies and immunoblotted with anti-phosphotyrosine antibodies.
