# Supplementary material for: The Structural Features of Trask That Mediate Its Anti-Adhesive Functions
Source: PLoS One. 2011 Apr 29;6(4):e19154. doi: 10.1371/journal.pone.0019154 (PMC3084758; doi:10.1371/journal.pone.0019154)
Supplement: Figure S2 — On the following pages several different views are shown for each cell type with/without doxycycline induction. The high magnification view is shown to best demonstrate morphologic characteristics while the low magnification view is shown to best demonstrate the generality of the adhesion phenotype across a larger cell population with much less selectivity due to the wider field of view. The microscopic images in this figure are from different clones than the ones shown in the main paper. This is to show consistency and account for effects related to clonal variability. (PDF) [file pone.0019154.s002.pdf]

Figure S2

Additional phase contrast microscope views of MDA-468TR transfectants

On the following pages several different views are shown for each cell type with/without doxycycline induction. The high magnification view is shown to best demonstrate morphologic characteristics while the low magnification view is shown to best demonstrate the generality of the adhesion phenotype across a larger cell population with much less selectivity due to the wider field of view.

The microscopic images in this figure are from different clones than the ones shown in the main paper. This is to show consistency and account for effects related to clonal variability.

magnification index

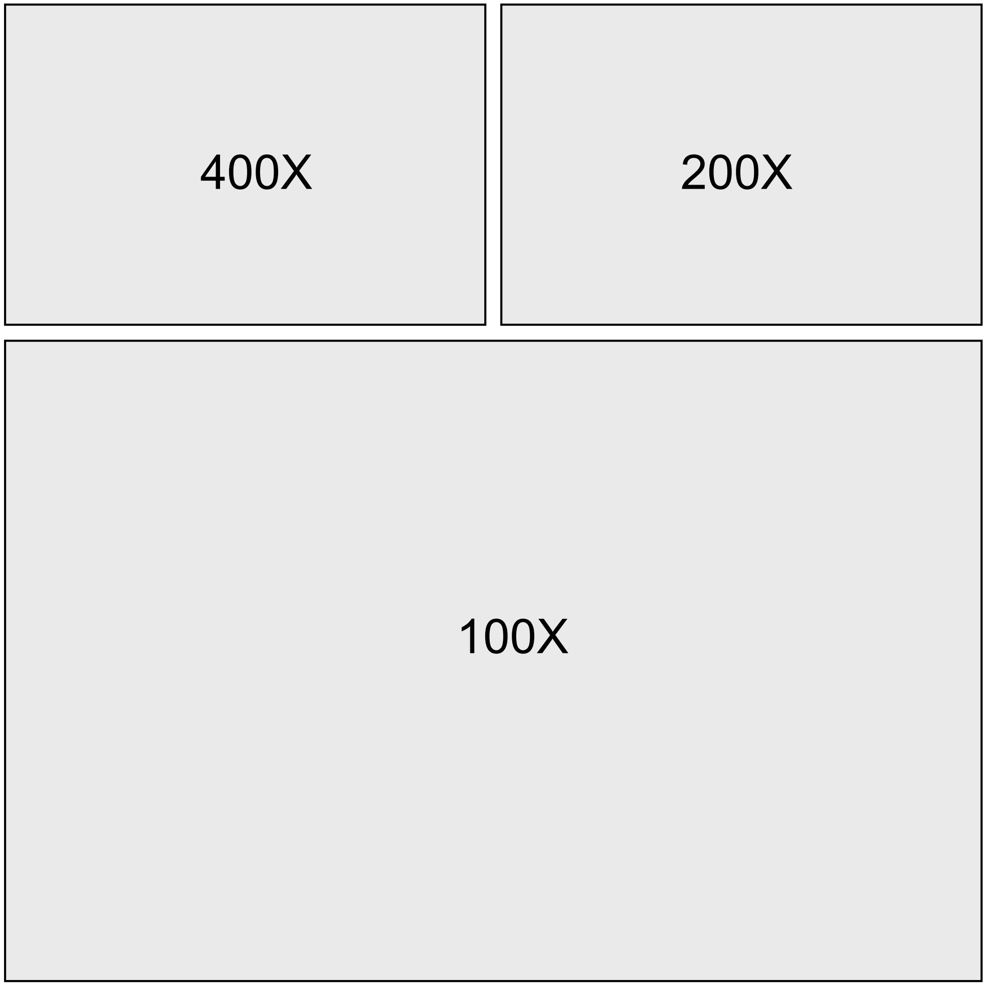

MDA-468TR/vector

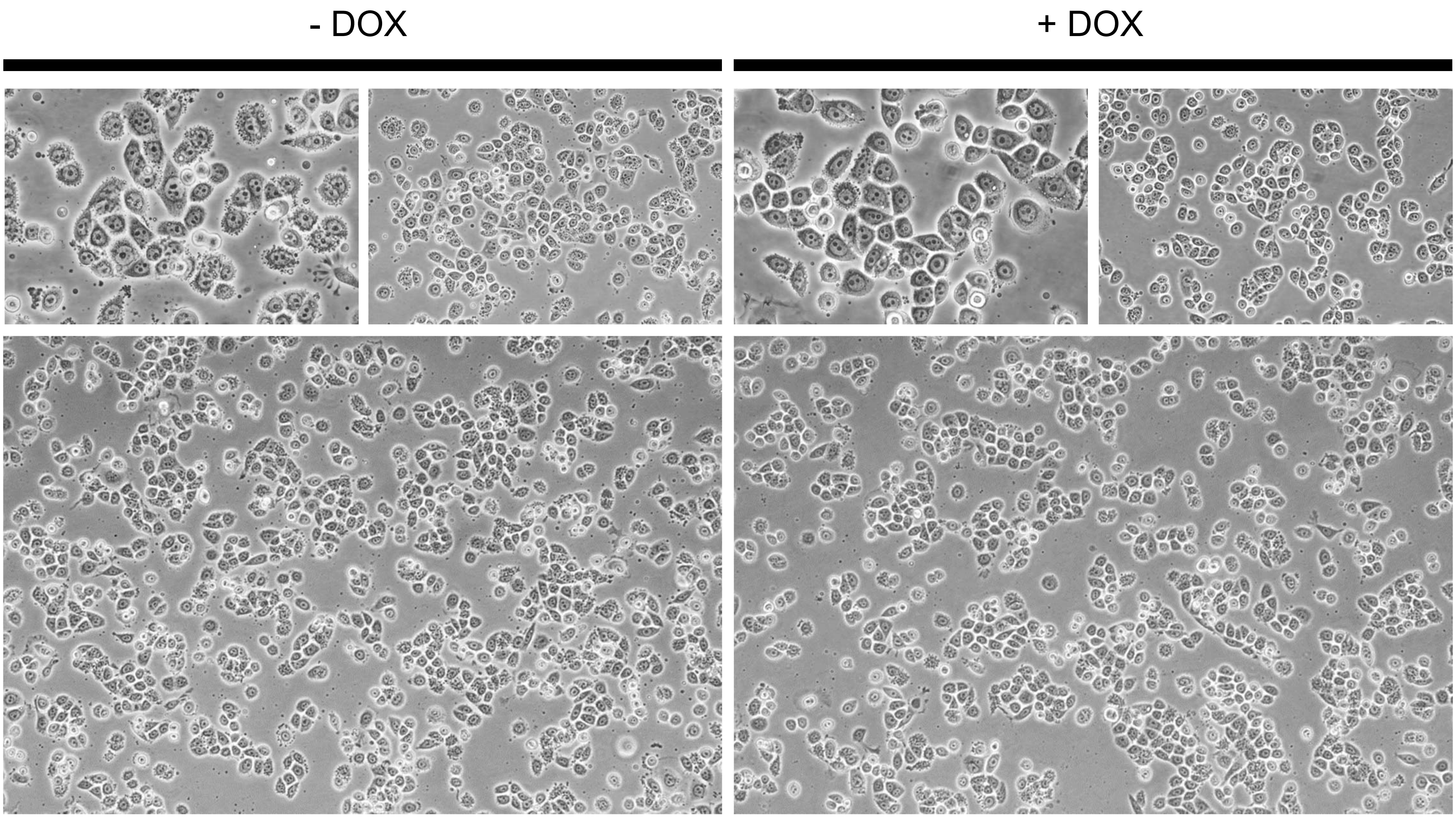

MDA-468TR/wt-Trask

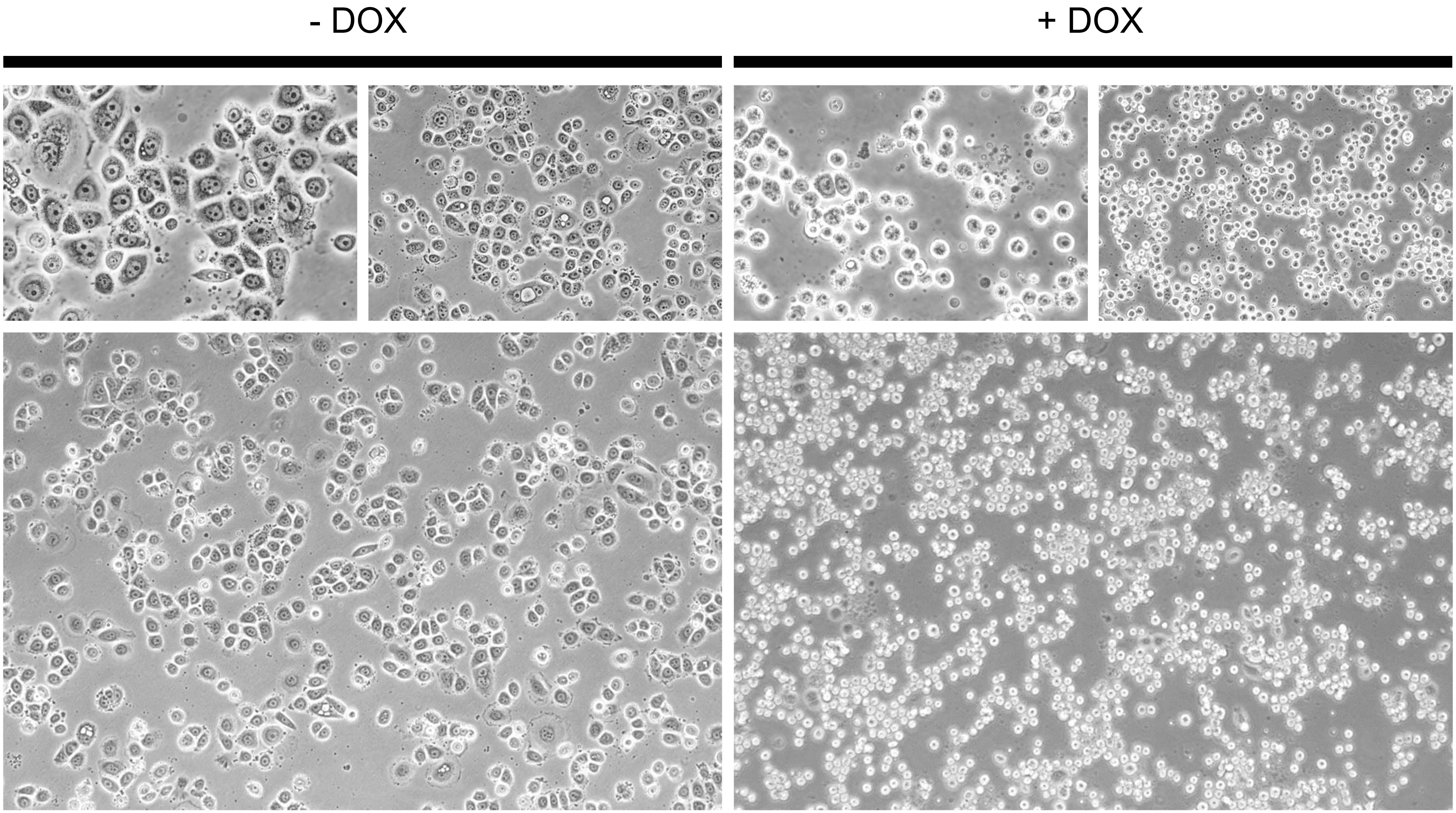

MDA-468TR/M4-Trask

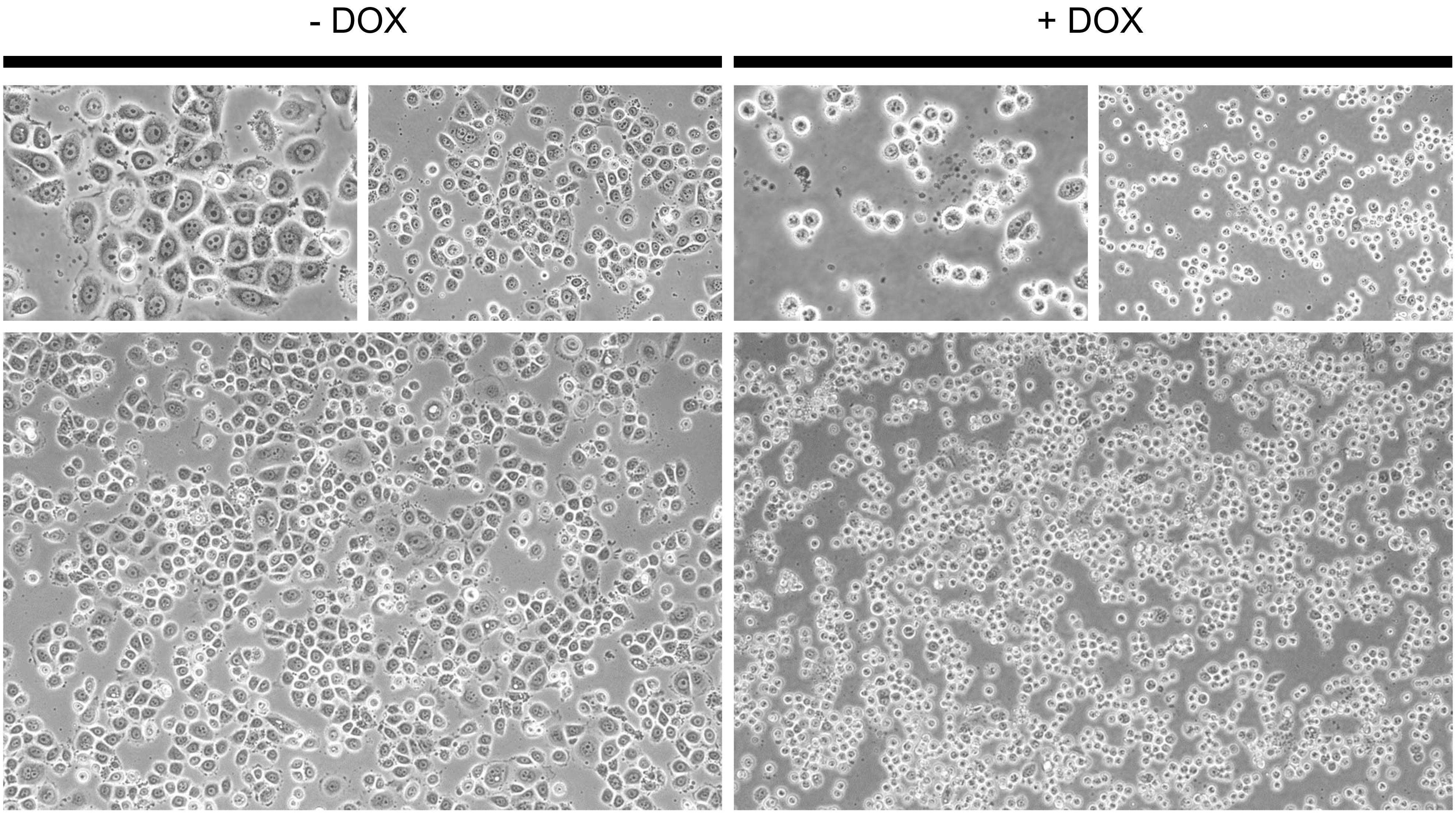

MDA-468TR/M5-Trask

- DOX + DOX

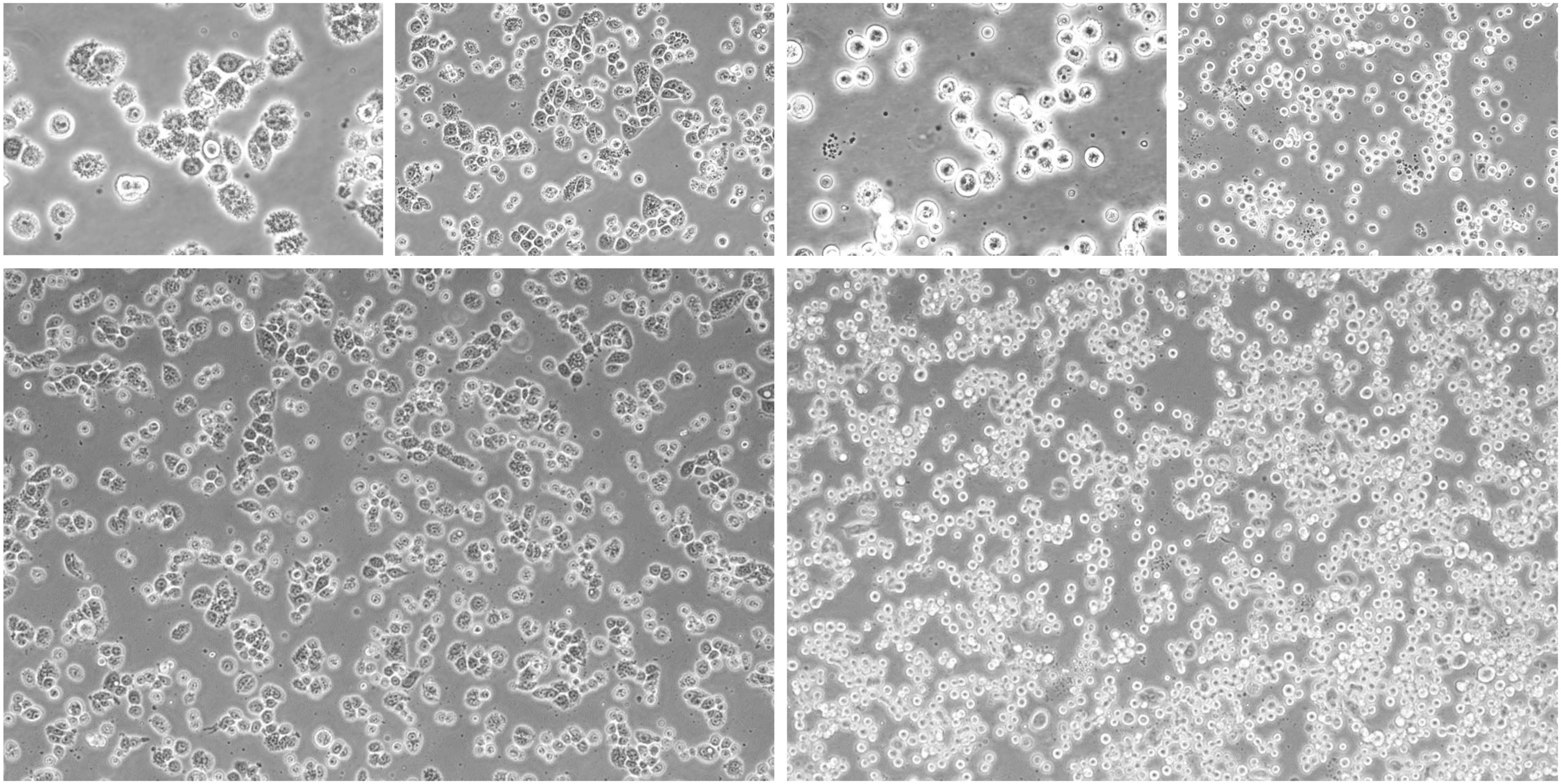

MDA-468TR/M7-Trask

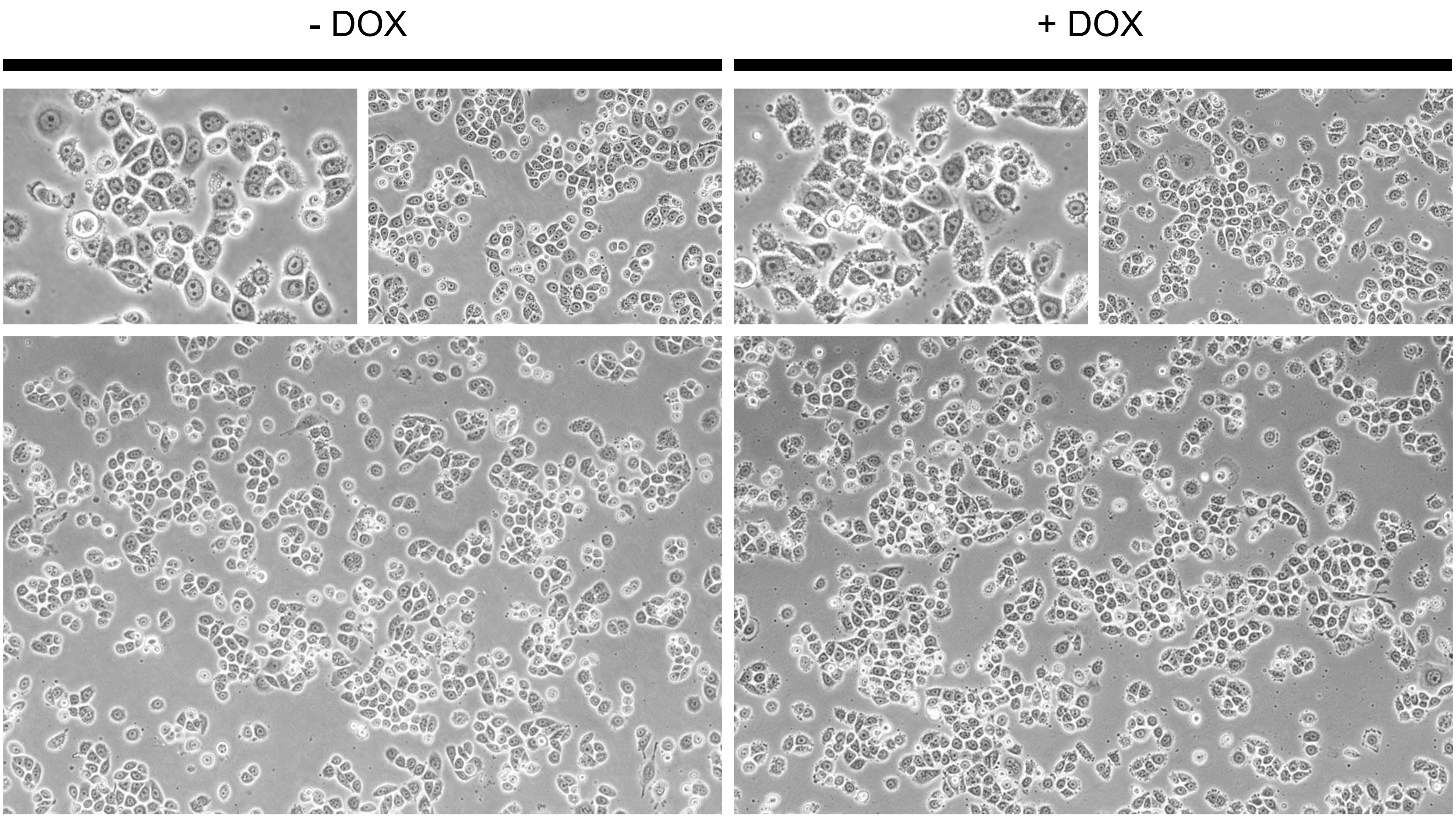

MDA-468TR/M8-Trask

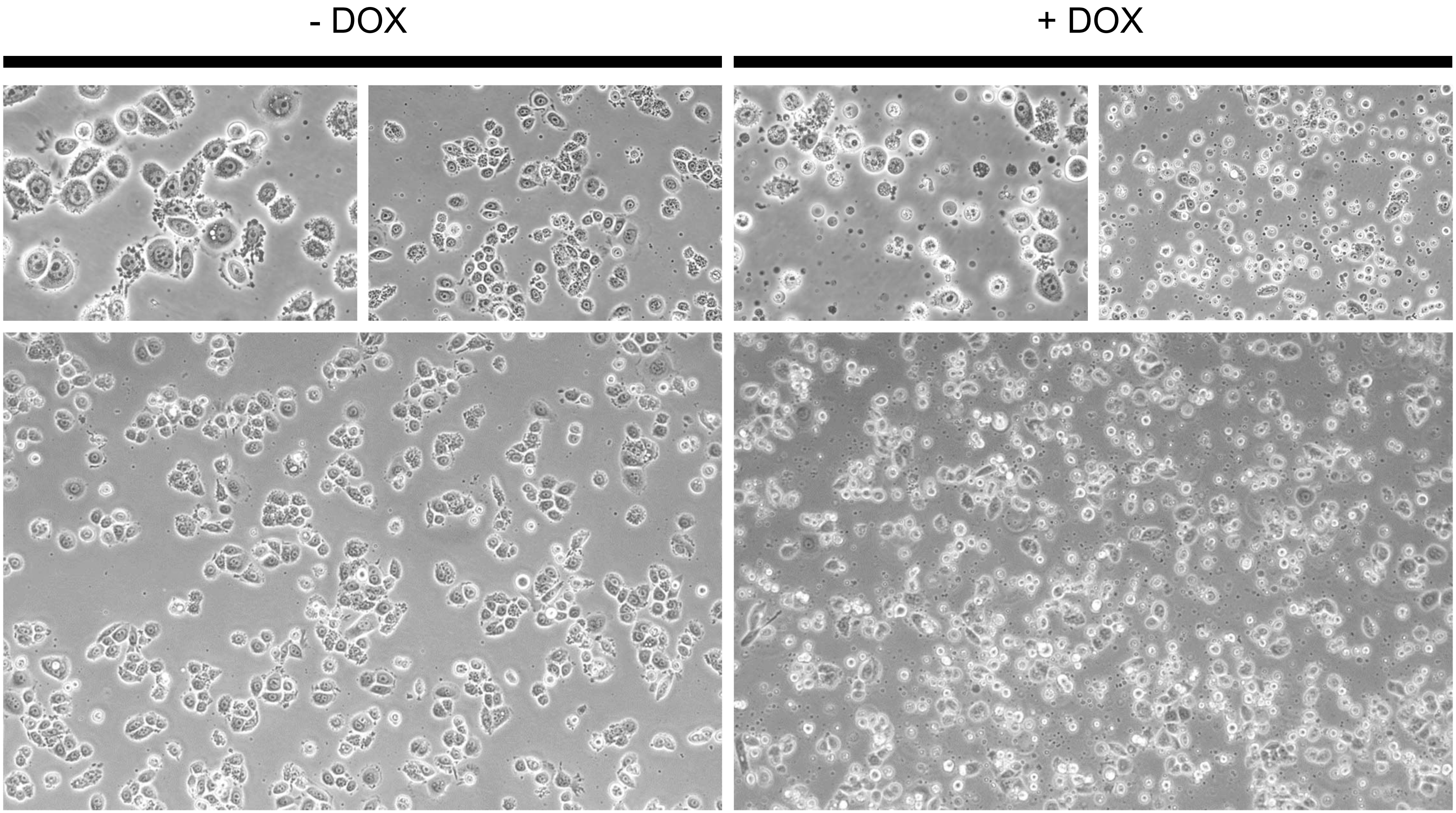

MDA-468TR/M9-Trask

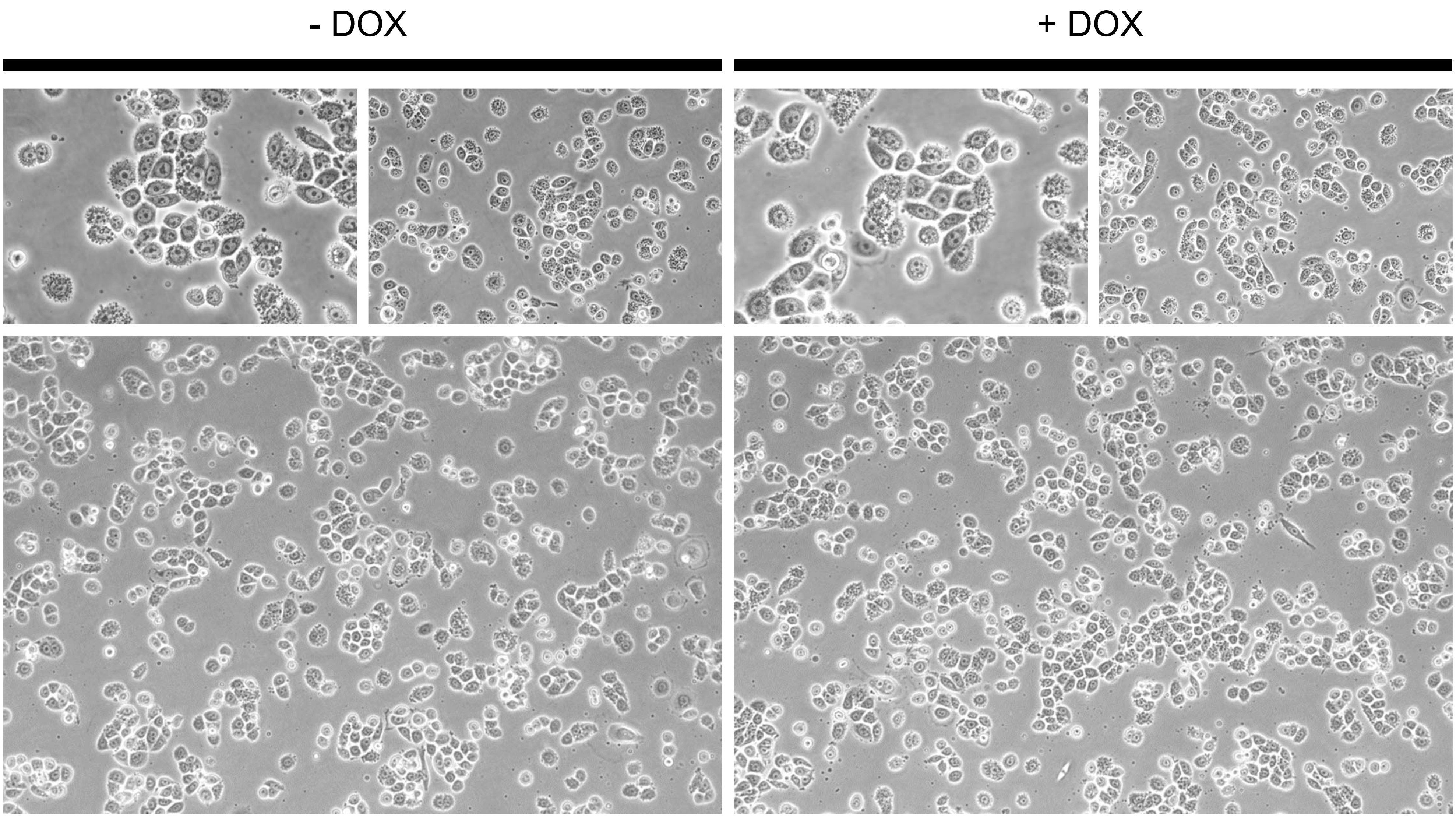

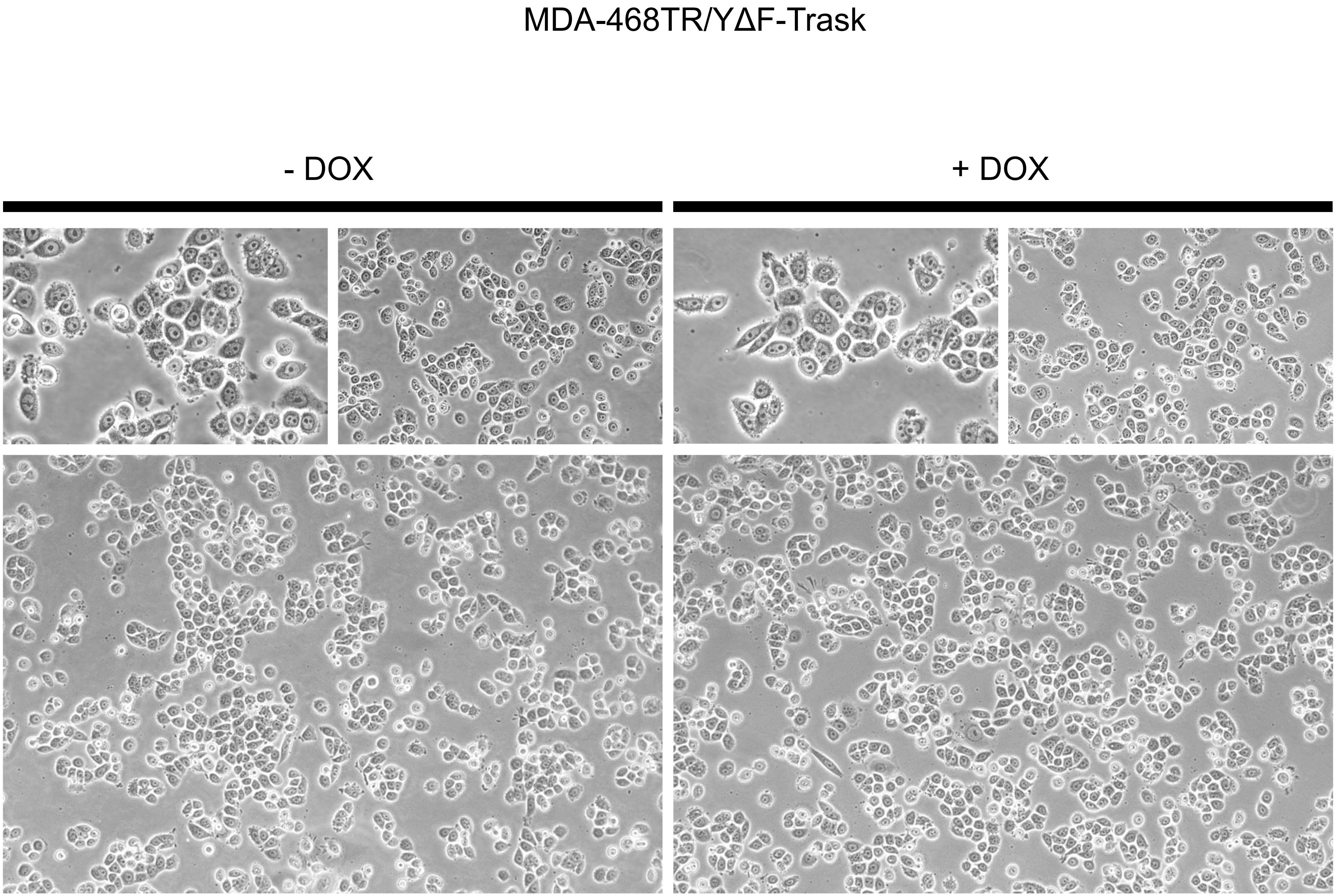
MDA-468TR/YΔF-Trask
- DOX
+ DOX
