# Supplementary material for: The Structural Features of Trask That Mediate Its Anti-Adhesive Functions
Source: PLoS One. 2011 Apr 29;6(4):e19154. doi: 10.1371/journal.pone.0019154 (PMC3084758; doi:10.1371/journal.pone.0019154)
Supplement: Figure S3 — The FAK phosphorylation data of Figure 4C was quantified by densitometry using the ImageJ program. The analysis was done separately for each paired data set (+/− doxycycline) corresponding to the different transfected cell types and normalized with respect to the uninduced (-dox) state. The data was not normalized with respect to total FAK expression, as a direct effect of the induction on FAK expression cannot be ruled out. (PDF) [file pone.0019154.s003.pdf]

Figure S3

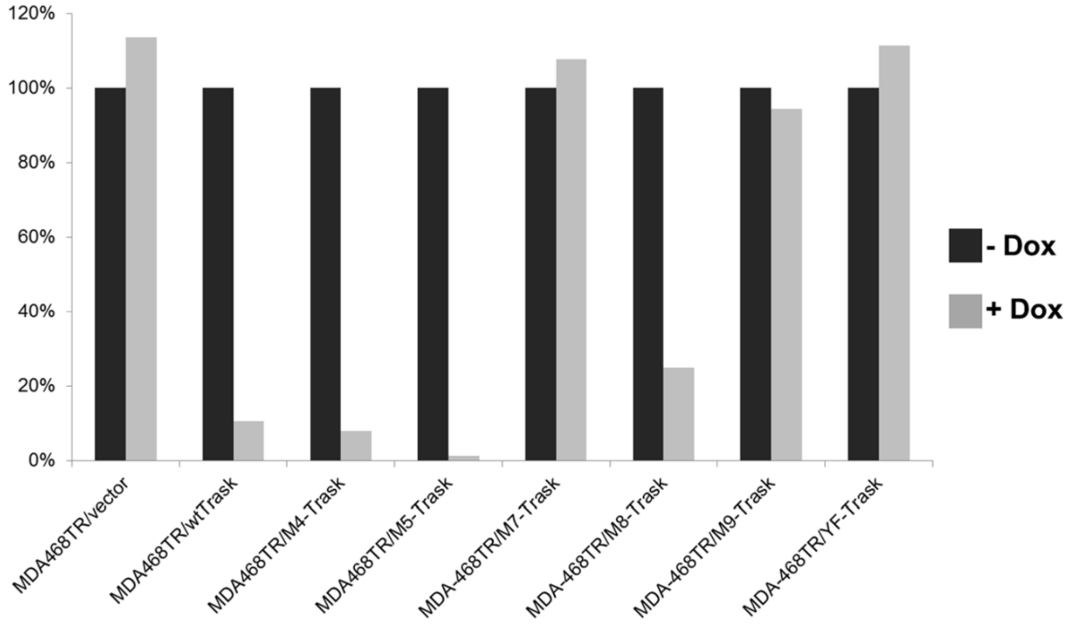

Figure S3: The FAK phosphorylation data of figure 4C was quantified by densitometry using the ImageJ program. The analysis was done separately for each paired data set (+/- doxycycline) corresponding to the different transfected cell types and normalized with respect to the uninduced (-dox) state. The data was not normalized with respect to total FAK expression, as a direct effect of the induction on FAK expression cannot be ruled out.
