# Supplementary material for: The Structural Features of Trask That Mediate Its Anti-Adhesive Functions
Source: PLoS One. 2011 Apr 29;6(4):e19154. doi: 10.1371/journal.pone.0019154 (PMC3084758; doi:10.1371/journal.pone.0019154)
Supplement: Figure S4 — MDA-468 cells were transiently transfected with the pcDNA4 vector or vector expressing the wildtype, M10, or M11 Trask mutants. Cell lysates were assayed as indicated. These are independent repeats of the experiment shown in the manuscript. (PDF) [file pone.0019154.s004.pdf]

Figure S4

Additional experiments with the M10 and M11 mutant constructs

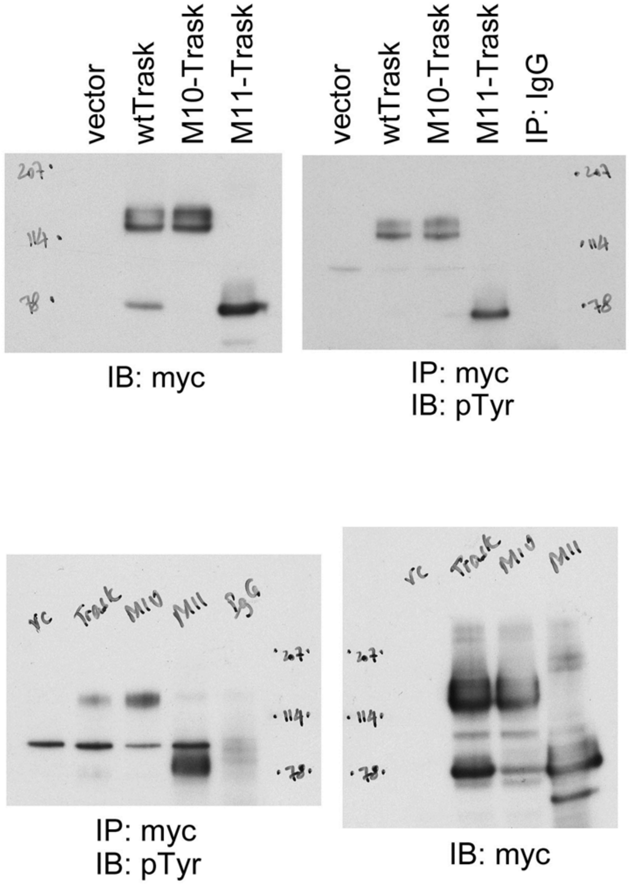

Figure S4: MDA-468 cells were transiently transfected with the pcDNA4 vector or vector expressing the wildtype, M10, or M11 Trask mutants. Cell lysates were assayed as indicated. These are independent repeats of the experiment shown in the manuscript.
